# Noninvasive Temperature Measurements in Tissue-Simulating Phantoms Using a Solid-State Near-Infrared Sensor

**DOI:** 10.3390/s24123985

**Published:** 2024-06-19

**Authors:** Ariel Kauffman, John Quan Nguyen, Sanjana Parthasarathy, Mark A. Arnold

**Affiliations:** 1 Rockley Photonics Inc., Irvine, CA 92614, USA; 2 Department of Chemistry, University of Iowa, Iowa City, IA 52242, USA

**Keywords:** body temperature sensing, near-infrared spectroscopy, wearable technologies, silicon photonic integrated chip, temperature, tissue phantoms

## Abstract

The monitoring of body temperature is a recent addition to the plethora of parameters provided by wellness and fitness wearable devices. Current wearable temperature measurements are made at the skin surface, a measurement that is impacted by the ambient environment of the individual. The use of near-infrared spectroscopy provides the potential for a measurement below the epidermal layer of skin, thereby having the potential advantage of being more reflective of physiological conditions. The feasibility of noninvasive temperature measurements is demonstrated by using an in vitro model designed to mimic the near-infrared spectra of skin. A miniaturizable solid-state laser-diode-based near-infrared spectrometer was used to collect diffuse reflectance spectra for a set of seven tissue phantoms composed of different amounts of water, gelatin, and Intralipid. Temperatures were varied between 20–24 °C while collecting these spectra. Two types of partial least squares (PLS) calibration models were developed to evaluate the analytical utility of this approach. In both cases, the collected spectra were used without pre-processing and the number of latent variables was the only optimized parameter. The first approach involved splitting the whole dataset into separate calibration and prediction subsets for which a single optimized PLS model was developed. For this first case, the coefficient of determination (R^2^) is 0.95 and the standard error of prediction (SEP) is 0.22 °C for temperature predictions. The second strategy used a leave-one-phantom-out methodology that resulted in seven PLS models, each predicting the temperatures for all spectra in the held-out phantom. For this set of phantom-specific predicted temperatures, R^2^ and SEP values range from 0.67–0.99 and 0.19–0.65 °C, respectively. The stability and reproducibility of the sample-to-spectrometer interface are identified as major sources of spectral variance within and between phantoms. Overall, results from this in vitro study justify the development of future in vivo measurement technologies for applications as wearables for continuous, real-time monitoring of body temperature for both healthy and ill individuals.

## 1. Introduction

Wearable technologies represent a growing segment of the consumer market that focuses on wellness and fitness [1,2]. The attractiveness of these devices is the ability to monitor and record important health-related physiological biomarkers continuously and in real-time. The measurement of heart rate, heart rate variability, respiration rate, and blood oxygen saturation (SpO_2_) are widely available on commercial smartwatches and fitness trackers. In general, heart rate, heart rate variability, and respiration rate are derived from analysis of a photoplethysmography (PPG) signal [3,4]. The PPG signal is determined from diffuse reflectance measurements associated with a band of green light with a peak emission at 524 nm. The resulting time-dependent PPG waveform includes a pulsatile signal derived from cardiovascular-induced changes in the volume of blood within the optical path. SpO_2_ measurements use pulse oximetry to measure the amount of oxygenated hemoglobin relative to the total hemoglobin in pulsating blood [5]. Both PPG and SpO_2_ signals are noninvasive and are commonly incorporated into modern wearable technologies.

Body temperature is another attractive biomarker for wellness and fitness monitoring [6]. Current technologies for monitoring body temperature for wellness and fitness applications rely on the measurement of skin surface temperatures. While skin surface temperatures are easy to measure, the relationship between the skin’s surface and core body temperatures is not straightforward [7,8]. Converting a skin surface measurement to a core body temperature demands knowledge of the heat transfer processes between (1) the core body volume and the skin surface, and (2) the skin surface and the ambient surroundings. A variety of heat transfer models are available to account for surface-to-environment heat flux when coupled with the so-called double sensor experimental configuration, originally proposed by Gunga and co-workers [7,8,9]. A more sophisticated heat transfer model, recently proposed by Shan et al. [10], accounts for the effects of blood perfusion within the tissue matrix. For general wellness and fitness applications, such heat transfer models must be accurate across both a diverse population as well as a myriad of ambient conditions.

Noninvasive spectroscopic sensing offers an alternative approach, whereby the body’s temperature is derived from optical measurements that interrogate below the epidermal layer of skin. This type of sub-surface measurement offers the potential advantage of reducing the impact of the ambient environment, thereby reducing the complexity of relating the measured temperature to the core body temperature. The utility of sub-surface temperature measurements was suggested recently in a white paper released by Rockley Photonics [11]. In this preliminary work, noninvasive temperatures derived from sub-surface near-infrared skin measurements showed superior correlations with respect to core body temperature in comparison to conventional thermometers associated with sublingual, tympanic membrane, and skin-surface forehead measurements.

Near-infrared spectroscopy has been explored by others to determine its ability to measure the temperature of aqueous environments based on temperature-dependent shifts in the spectrum of water over near-infrared wavelengths. Lin and Brown [12] envisioned a fiber-optic temperature sensor and reported measurements performed on pure water with defined optical path lengths. Their data illustrate linearity between the position of a water absorption band and temperature. Multivariate calibration models resulted in standard error of prediction (SEP) values ranging from 0.2 to 0.6 °C, over a temperature range of 5–85 °C. Andrade et al. [13] reported multivariate temperature models for aqueous solutions with different ionic strengths and pH. These models displayed SEP values of 0.2–0.5 °C, over a temperature range of 15–90 °C. Work published by Kakuta et al. [14] demonstrated the utility of using near-infrared spectroscopy to determine temperatures of aqueous mixtures composed of 2–3% Intralipid in water. Models based on linear regression and partial least squares (PLS) regression provided prediction errors of 0.2–0.3 °C and ≤0.2 °C, respectively. Finally, Small and his colleagues [15] characterized PLS calibration models for temperature measurements in a variety of sample matrixes composed of aqueous solutions with different pHs, ionic strength, and levels of glucose, lactate, urea, bovine plasma, and human serum albumin. SEP values of 0.2–0.4 °C were obtained based on spectra collected over an extended period of 13 months, using a pre-processing strategy involving a standard normal variate transform combined with a discrete wavelet transform.

The previous work mentioned above focused on temperature determination in liquid-phase aqueous-based samples which differ significantly from the complex, solid matrix of human skin. Such drastic sample differences can have impacts on spectral complexity and variation, spectral processing, and model complexity, as well as important considerations regarding the introduction and collection of light during the actual measurement. The objective of the work presented here is to expand the sample complexity for near-infrared aqueous temperature measurements with an eye towards noninvasive measurements of human skin. With this application in mind, a prototype solid-state laser-based spectrometer was used to collect near-infrared spectra from a set of phantoms designed to simulate the dermal layer of skin. In total, seven tissue-simulating phantoms were prepared with different levels of gelatin protein, Intralipid, and water, and phantom temperatures ranged between 20 and 24 °C.

## 2. Materials and Methods

### 2.1. Instrumentation and Data Collection

Spectra were collected using an early-stage prototype of a solid-state laser spectrometer consisting of a multi-wavelength, repetitively pulsed laser array spanning the short wavelength range of the near-infrared spectrum. The spectroscopic sensing module consisted of a monolithically integrated silicon photonic integrated chip featuring a set of 27 III-V semiconductor lasers. The source optics were designed to pass 99% of the radiation captured from each laser through a sample path and the remaining 1% of this radiation passes through an internal reference path. For the sample path, radiation from each laser was directed to the phantom matrix, and a portion of the diffuse reflected radiation from the illuminated phantom matrix was detected by a sample photodiode. The intensity of the 1% radiation of the internal reference path was measured by a separate photodiode.

Diffuse reflectance spectra were collected by pressing the spectrometer sensing module against the surface of the tissue phantom. For data collection, lasers were pulsed individually for 200 μs and both the diffuse reflectance and internal reference intensities were recorded. On either side of each laser pulse, a dark signal was collected with the laser turned off, resulting in a per laser pattern of “off-on-off”. This pattern was repeated for each laser wavelength and the resulting modulation cycle was processed to produce a single-beam spectrum.

### 2.2. Tissue-Simulating Phantom Construction

Tissue-simulating phantoms were prepared by dissolving a known mass of gelatin (Knox) into a volume of 50 °C de-ionized type II water (Grainger). The gelatin was added gradually with constant stirring with a magnetic stir bar rotating at a rate of 200 revolutions per minute (rpm). The stir rate was increased to 220 rpm while maintaining the temperature at 50 °C until all gelatin was dissolved, as determined by visual inspection. The temperature was then decreased to 40 °C before 25 mL of 20% Intralipid emulsion (Sigma-Aldrich Chemical Company) was added. The mixture was returned to the hot plate and heated to 45 °C while stirring at a rate of 200 rpm.

**Figure 1:**
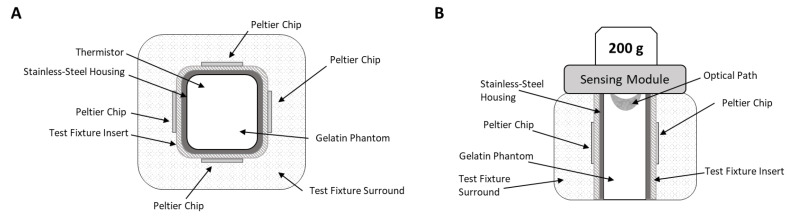
Schematic diagram of the measurement configuration showing a top view (**A**) and side view (**B**) of the sample holder. The side view includes the positioning of the sensing module relative to the surface of the tissue-simulating phantom.

The resulting mixture was then transferred to a custom stainless-steel fixture, as shown schematically in Figure 1. Any visible bubbles were removed by using a transfer pipette and the fixture was sealed with a custom Plexiglas lid secured by screws at the four corners. Upon completion, the phantoms were placed in a 4 °C refrigerator to cool for at least 30 min prior to measurements. The Plexiglas lid was removed before collecting spectra. In total, seven phantoms were prepared and the composition of each is provided in Table 1.

The combinations of gelatin protein, lipid scatterers, and water, along with their proportions, were selected to mimic the major near-infrared absorbers in human skin. In addition, our formulation was intended to match the skin’s scattering properties. Still, the chemical matrix, for example, is much simpler compared to human skin and issues related to changes in the refractive index of the extracellular space are not modelled by this phantom matrix. Most significantly, the basic formulation used here limits experiments to temperatures below 24 °C in order to maintain the phantom’s solid, non-liquid structure.

### 2.3. Temperature Control and Collection of Spectra

The test fixture noted above, and shown schematically in Figure 1, was designed to control the temperature of the phantom matrix. Temperature control was achieved with a set of four thermoelectric Peltier chips positioned on each face of the cube-shaped fixture, as shown in Figure 1A. Each chip was set at the desired temperature, as listed in Table 1, and the temperature of the phantom matrix was recorded in real-time using a wire thermistor positioned 1 to 2 mm below the phantom’s surface. As indicated in Figure 1B, a 200 g mass was placed on top of the sensing module to aid in maintaining direct contact between the spectrometer and the surface of the tissue phantom.

For each phantom, spectral data were collected over a unique pattern of five temperature steps over a range between 20 and 24 °C. After thermally equilibrating the phantoms with the ambient room temperature, each of the four Peltier chips was set to the first target temperature, as indicated in Table 1. Both spectral and temperature data were recorded simultaneously and continuously. As a result, spectra were collected at temperatures between the designated temperature steps as the phantom matrix adjusted to the new Peltier chip temperature. For each spectrum, the assigned temperature was determined by a linear interpolation between the two temperature readings that bracketed the spectrum in time.

A total of twenty 10-s measurements were recorded at a data collection frequency of 15.7 hertz, resulting in a total of 157 spectra collected for each 10 s recording. Each recording was separated by 50 s of “off” time during which no spectral data were collected. Each recording plus off-time lasted 60 s in duration, resulting in a total measurement time of twenty minutes per temperature step. The temperature step pattern is specified in Table 1 for each tissue phantom. The matrix composition limited the maximum temperature of the study to 24 °C. At higher temperatures, the gelatin matrix began to liquefy, thereby resulting in a decoupling of the phantom surface and the spectrometer sensing module.

### 2.4. Data Processing

Each modulation cycle produced 84 data points, 3 data points per laser (off-on-off), and 3 off measurements at the end of each cycle. For each wavelength, the measured sample intensity was calculated as the photodiode signal for the laser’s on-signal minus the average dark signal from the corresponding laser’s off-signals collected immediately before and after the on-pulse, according to Equation (1). This process converts the 84-point modulation cycle to a 27-wavelength single-beam spectrum.

(1) $$V_{Laser}=V_{LaserOn}-mean(V_{LaserOff1},V_{LaserOff2})$$

As noted above, approximately 1% of the incident radiation for each laser was captured as an internal reference measurement. Each internal reference measurement was corrected for the dark signal, as described above in Equation (1) and this dark-signal-corrected reference signal was further adjusted to account for differences in both detector gains and incident radiation powers between the sample (diffuse reflectance) and the internal reference channels. The final internal reference signal corresponded to an air reference single-beam spectrum and served to correct for real-time power fluctuations during operation. Absorbance spectra were calculated as the negative base-10 logarithm of the ratio of a sample single-beam spectrum relative to the corrected internal air reference single-beam spectrum.

Data acquisition and temperature control of the phantoms were achieved using custom software developed in-house. All data analyses were performed using MATLAB R2021b (MathWorks, Natick, MA, USA). All machine learning routines were written in-house.

## 3. Results and Discussion

### 3.1. Spectral Range

Molecular spectroscopy across the near-infrared spectrum consists of multiple orders of overtone and combination transitions associated with vibrational modes for selected molecular bonds. The vibrational spectroscopy of water strongly influences the underlying spectral features observed in aqueous solutions owing to the high concentration of infrared-active O-H bonds. Hydrogen bonding modulates the absorptivities of the O-H bond, thereby resulting in a strong temperature sensitivity of the water spectrum over the near-infrared wavelengths. As noted above, numerous research groups have demonstrated the potential of measuring the temperature of aqueous solutions from an analysis of near-infrared spectra. Most notably, the principal water absorption bands across the near-infrared spectrum display a prominent blue-shift (a shift toward higher energy) with increasing temperatures.

The work summarized in the Introduction section featured different regions of the near-infrared spectrum for temperature measurements. Andrade and co-workers [13] used spectra collected over the 600–1100 nm spectral range (16,700–9090 cm^−1^), corresponding to the second order of the water O-H stretching vibration. Temperature models presented by both Brown and Kakuta [12,14] relied on spectral information collected over the first overtone of the water O-H stretching vibration mode (ca. 1300–1620 nm or 7690–6170 cm^−1^). Small and his research team [15] constructed temperature calibration models based on near-infrared spectra collected over the range of 2130–2380 nm (4700–4200 cm^−1^). This spectral range sits between the fundamental water O-H stretch centered at 2780 nm (3600 cm^−1^) and the first combination of the O-H stretch and H-O-H bending modes centered at 1920 nm (5200 cm^-1^). The range used by Small’s group is an absorption minimum for aqueous solutions and features first-order combination bands associated with C-H, N-H, and O-H bonds in organic molecules. In combination, these studies illustrate how multiple spectral regions over the near-infrared spectrum are available for temperature measurements in aqueous environments.

In this work, absorbance spectra were collected over a set of proprietary wavelengths associated with the first overtone O-H stretch of water. This spectral band was selected on the basis of analytical sensitivity. In comparison, the absorptivity of the first-order O-H water band is approximately ten-fold higher than that of the second-order O-H water band, thereby providing higher measurement sensitivity [16]. In addition, the shorter wavelengths of the first overtone spectral region offer deeper penetration depths in comparison to the longer wavelengths of the combination band. Deeper penetration depths enhance sensitivity because of longer effective aqueous path lengths.

### 3.2. Spectral Sensitivity to Temperature

Principal component analysis (PCA) was performed to assess the relationship between spectral variance and the temperature of the phantom matrix. Accordingly, a PCA calculation was performed on the full set of 700 tissue phantom spectra (seven phantoms × five temperatures × twenty spectra/temperatures). The spectra used for this PCA calculation corresponded to the raw absorbance spectra collected for each phantom without any prior spectral pre-processing.

**Figure 2:**
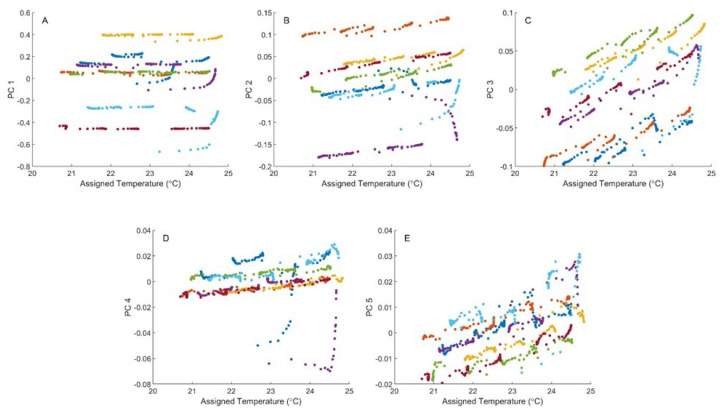
PCA score–temperature plots for raw absorbance spectra collected at different temperatures for Phantom 1 (blue), Phantom 2 (orange), Phantom 3 (yellow), Phantom 4 (purple), Phantom 5 (green), Phantom 6 (cyan), and Phantom 7 (red). Score–temperature plots are provided for the first five principal components, designated as PC 1 (**A**), PC 2 (**B**), PC 3 (**C**), PC 4 (**D**), and PC 5 (**E**).

The five score–temperature plots presented in Figure 2 summarize the PCA results. For each score plot, the principal component scores are plotted relative to the temperature assignment to each spectrum. The plotted points are color-coded to distinguish each phantom, as specified in Table 1. These first five principal components cumulatively explain 99.87% of the spectral variance throughout this tissue phantom spectral dataset.

The plots presented in Figure 2 illustrate a strong relationship between spectral variance and the assigned phantom temperature. Although the scores plotted for the first principal component are relatively insensitive to sample temperature, significant correlations are evident between temperature and principal component scores for the second, third, fourth, and fifth principal components. Such correlations support the feasibility of multivariate calibration models for the measurement of phantom temperature.

Figure 2 also reveals several periods with extensive jumps in the principal component scores, particularly for Phantoms 1 (blue), 3 (yellow), 4 (purple), and 6 (cyan). This type of spectral variance is especially prominent in the second principal component score plots for Phantoms 3, 4, and 6, the third principal component plots for Phantoms 3 and 6, and the fourth principal component score plots for Phantoms 1 and 4. These types of variations correspond to sudden changes in the measured diffuse reflectance signal and are attributed to within-run instability of the interface between the sensing module and the phantom surface. Such instabilities constitute systematic errors, thereby justifying the exclusion of these data points as outliers from our analytical analyses. Of the 100 data points collected for each Phantom, nine, five, twenty, and fourteen outlier points were removed from Phantoms 1, 3, 4, and 6, respectively. Overall, the dataset size was reduced from 700 to 652 spectra, corresponding to a 93.1% retention. The removed data points are identified in Figure S1, which shows the same five principal component score plots presented in Figure 2, but with a box encompassing each of the removed outlier data points.

**Figure 3:**
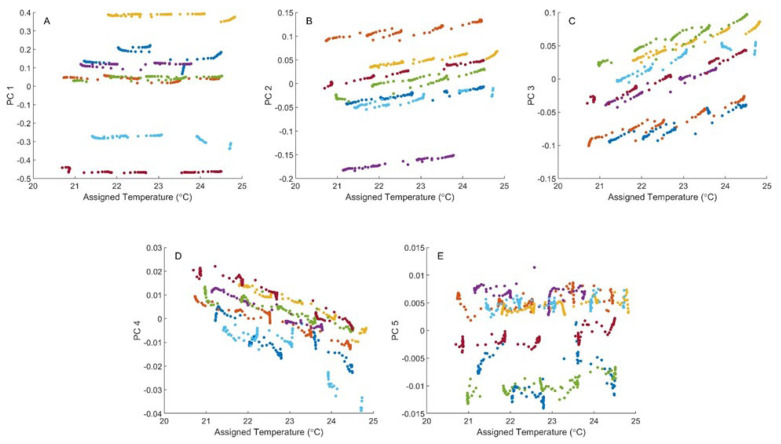
PCA score–temperature plots for raw absorbance spectra with outliers removed. See caption of Figure 2 for details.

The PCA was repeated after removing the outlier spectra and the resulting score–temperature plots are presented in Figure 3. This second set of scores is similarly distributed to the one displayed in Figure 2 for the first, second, and third principal components. In contrast, major differences are noted for the fourth and fifth principal component plots in Figure 2 and Figure 3. For principal component 4, the score–temperature plots in Figure 3 have a negative slope compared to the moderate positive slopes observed in Figure 2. Figure 3 also illustrates that the PCA scores for the fifth principal component are insensitive to temperature.

The score–temperature data plotted in Figure 3 were fit to a linear function for each phantom. These fits provide a measure of the sensitivity of scores to temperature (slope), offsets between phantoms (y-intercepts), and goodness of fit within each phantom experiment (coefficient of determination, R^2^). Results for the various regression calculations are summarized in Table S1.

As expected, the regression slopes are low and the correlations (R^2^) between scores and temperature are poor for the first and fifth principal components. The largest slopes are observed for the second, third, and fourth principal components and, likewise, correlations between scores and temperature are strongest for these principal components (2nd, 3rd, and 4th). The range of R^2^ values across the seven phantoms are 0.87–0.99, 0.88–0.98, and 0.66–0.96 for principal components 2, 3, and 4, respectively.

Offsets are also evident in the score plots presented in Figure 3. Spectral offsets between phantoms are most apparent in the plots for the first, second, and third principal components. Likewise, the relative variances of the y-intercepts listed in Table S1 are higher for the first, second, and third principal components relative to the fourth and fifth. Relative variance values are 3.6, 0.6, 1.3, 0.007, and 0.03 A, respectively, for the five principal components represented in Figure 3. These values illustrate that the highest degree of offset variance is described by the first principal component.

**Figure 4:**
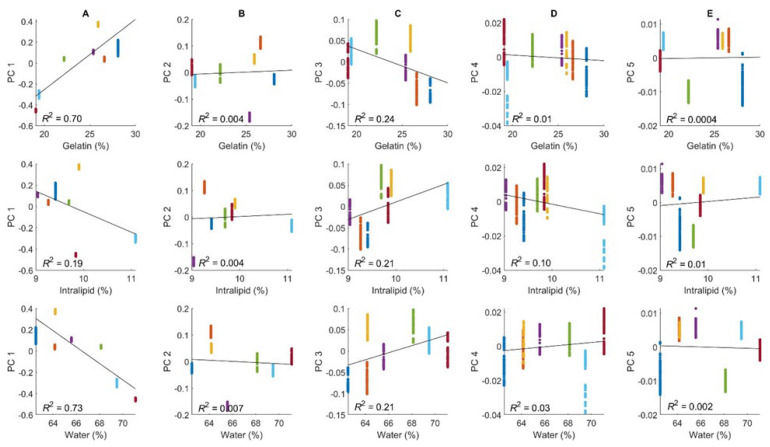
PCA score–chemical composition plots for prepared gelatin, Intralipid, and water of the tissue-simulating phantoms. Score–chemical composition plots are provided for the first five principal components, designated as PC 1 (**A**), PC 2 (**B**), PC 3 (**C**), PC 4 (**D**), and PC 5 (**E**). Solid lines represent linear fits. See caption of Figure 2 for details.

Differences in the chemical composition of the phantoms affect their absorption and scattering properties and could contribute to the observed spectral offsets. To visualize these effects, PCA scores were plotted as a function of the concentrations of water, gelatin, and Intralipid for the prepared phantoms. These plots are presented in Figure 4, and Table S2 summarizes the results of linear regression analysis for each of the five principal components. In general, the observed R^2^ values are low (ranging from 0.002 to 0.21) for all plots except for the first principal component scores versus the concentrations of water and gelatin. The R^2^ values for these water and gelatin plots are 0.73 and 0.70, respectively, indicating a correlation between spectral offsets and the chemical composition of the phantom. For comparison, the R^2^ value is only 0.19 for the Intralipid plot for the first principal component. The similarities in R^2^ for water and gelatin are caused by a strong concentration correlation for these two major matrix constituents (R^2^ = 0.98). Of course, uncontrolled variations associated with the environment, instrumentation, and sample interface may also be potential contributors to the observed spectral offsets.

### 3.3. Partial Least Squares Analysis

PLS calibration models were examined for the ability to predict the temperature of the tissue phantoms based on spectra collected with the prototype solid-state laser spectrometer. Two modeling strategies were explored. The first involved splitting the full dataset into separate calibration and prediction subsets. In the second case, a leave-one-phantom-out cross-validation strategy was used. As implemented, both methods are designed to test the ability of the model to predict temperatures from spectral data collected outside the calibration period.

#### 3.3.1. PLS Model from Calibration–Prediction Split

A single PLS calibration model was generated after splitting the dataset into calibration and prediction subsets. The spectral data collected for each phantom were separated into the spectra associated with the first three temperature steps and those associated with the final two temperature steps, as specified in Table 1. Spectra in the first grouping were used to construct the calibration model while those in the second grouping were used to judge prediction accuracy. This strategy aims to demonstrate the ability of the PLS model to predict temperatures accurately from spectra collected outside the calibration period across the seven phantoms. For a given phantom, the spectral features associated with temperature steps performed outside of the calibration period are represented by temperature steps associated with the other phantoms.

The full dataset used in this analysis corresponded to the 700 spectra collected across all seven phantoms, excluding the 48 outlier spectra described above. As a result, the calibration dataset was composed of 372 absorbance spectra and the prediction dataset was composed of 280 absorbance spectra. As noted in the Materials and Methods section, temperatures were assigned to each spectrum by linear interpolation between the two bracketing temperatures.

The PLS calculation used mean-centered spectra by phantom. No additional pre-processing was attempted to improve performance. The only optimized parameter was the number of latent variables, or factors, used in the final PLS calibration model. A leave-one-phantom-out cross-validation strategy was used for this optimization. In this calculation, all spectra in the calibration dataset associated with a given phantom were removed and a PLS calibration model was developed using the retained calibration spectra along with their assigned temperatures. Temperatures were then predicted for each spectrum of the left-out phantom. This process was repeated until each phantom had been left out once and, when completed, the standard error of cross-validation (SECV) was calculated for the predicted temperatures associated with the left-out phantom spectra. The entire process was repeated for 1–10 latent variables and the optimum number of latent variables was determined based on Akaike’s information criterion, which is used here to avoid over- or under-fitting by providing a metric for assessing the balance between goodness of fit and model complexity [17].

The following equation was then used to calculate the predicted temperature for the left-out spectra:

(2) $${\hat{c}}_{pred}=\left(A_{pred}-{\bar{A}}_{cal}\right)\;*\;b+{\bar{c}}_{cal}$$

where ${\hat{c}}_{pred}$ is the predicted temperature for a left-out spectrum, $A_{pred}$ is the left-out absorbance spectrum, ${\bar{A}}_{cal}$ is the average absorbance spectrum for the calibration dataset, b is the PLS regression vector, and ${\bar{c}}_{cal}$ is the average assigned temperature for the spectra in the calibration dataset. 

**Figure 5:**
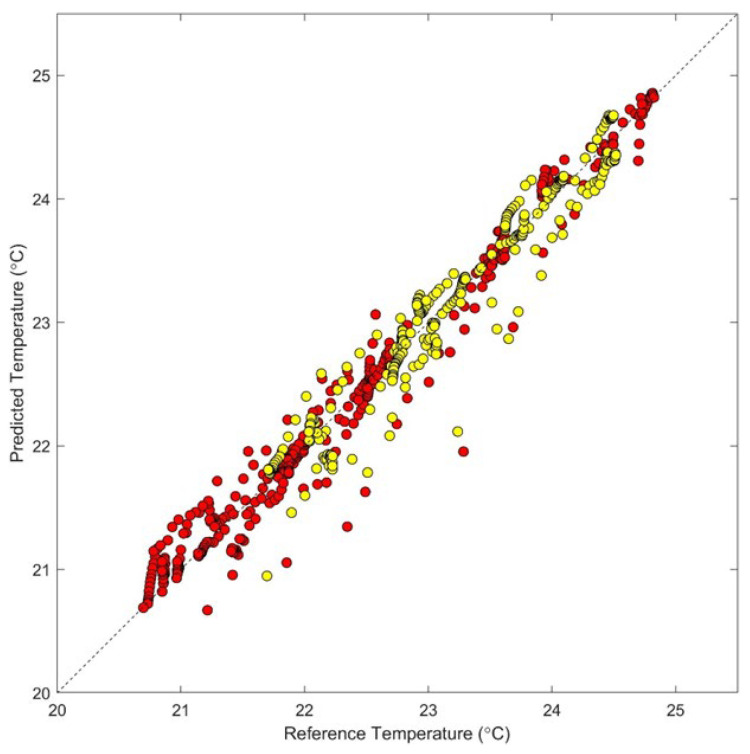
Temperature correlation plot showing results from the optimized 3-factor PLS calibration model based on splitting the dataset into a calibration dataset composed of spectra associated with the first three temperature settings and the prediction dataset composed of the remaining spectra for each phantom. Data plotted as red circles correspond to leave-one-phantom-out cross-validation points and the yellow circles correspond to predicted temperatures for the spectra in the prediction data set. The dashed line represents the ideal unity line.

Figure 5 shows the results from the optimized PLS model, which used three latent variables. In this figure, the predicted temperatures are plotted relative to the assigned temperatures for both the points used for calibration (red) and those used for prediction (yellow). In both cases, the calibration and prediction points align along the ideal unity line. Least squares linear fits provide a slope of 0.94 (±0.01), a y-intercept of 1.4 (±0.2) °C, and an R^2^ value of 0.95 for the 372 calibration data points. For the corresponding 280 prediction data points, a slope of 1.00 (±0.02), a y-intercept of −0.1 (±0.4) °C, and an R^2^ value of 0.94 were obtained.

Overall, the SECV is 0.26 °C, the standard error of prediction (SEP) is 0.22 °C, and the relative standard error of prediction (RSEP) is 0.94%, calculated according to Equations (3) and (4), where $c_{i}$ is the reference concentration for the sample, *i*, and ${\hat{c}}_{i}$ is the corresponding estimated concentration produced by the calibration model. The *n* − *h* − 1 term corresponds to the degrees of freedom for *n* samples and a model based on *h* independent variables with an intercept term.

(3) $$SECV=\sqrt{\frac{\sum {\left(c_{i}-{\hat{c}}_{i}\right)}^{2}}{n-h-1}}$$

(4) $$SEP=\sqrt{\frac{\sum {\left(c_{i}-{\hat{c}}_{i}\right)}^{2}}{n}}$$

The method used here splits the spectral data into separate calibration and prediction subsets according to when the spectra were collected. By using spectra collected from the first three temperature steps to predict the temperatures for the last two temperature steps, the model must have the ability to make accurate temperature predictions beyond the calibration period. In other words, extrapolated predictions are required. Still, the calibration and prediction spectra were collected at nearly the same time and extrapolations must only be accurate for a relatively short period beyond collection of the calibration data. For context, the data collected for each phantom were obtained over a period of 100 min (20 min per temperature step × five temperature steps), with the calibration data collected over the first 60 min and the prediction data collected over the next 40 min.

#### 3.3.2. Leave-One-Phantom-Out PLS Models

A more rigorous test of the method’s analytical utility is to predict temperatures for a set of phantom spectra completely omitted from the calibration process. Similar to the first method, extrapolated predictions are required but the prediction set represents a sample matrix not seen by the model but with the same temperature range as the calibration set. From the perspective of a wearable device, this scenario would represent a calibration model predicting data from a different user whereas the first method would represent the prediction of a user’s data following a baseline period and inclusion of that user’s data to the calibration model. For this purpose, a leave-one-phantom-out strategy was evaluated. Accordingly, all spectra for a given phantom were set aside for prediction purposes while all spectra associated with the remaining six phantoms were used to build the calibration function. By using this strategy, seven unique PLS calibration models were prepared and evaluated.

As before, the outlier spectra were omitted, and the same temperature assignments were used. All spectra used for calibration purposes were mean-centered by the phantoms prior to input into the PLS algorithm. No other pre-processing was performed. The optimum number of latent variables was determined by using the same leave-one-phantom-out cross-validation method described above, and predicted temperatures were obtained from Equation (2).

**Figure 6:**
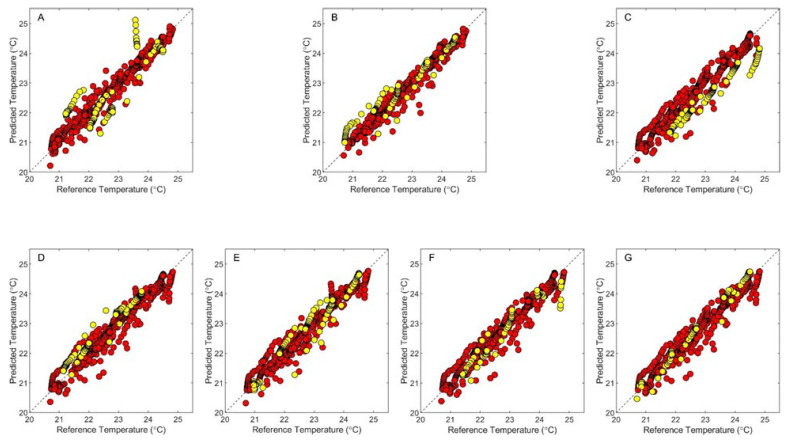
Temperature correlation plots showing results from the optimized PLS calibration models in which each phantom was left out of the calibration dataset in turn and utilized as the prediction data set. Plots are shown for models created when Phantoms 1 (**A**), 2 (**B**), 3 (**C**), 4 (**D**), 5 (**E**), 6 (**F**), and 7 (**G**) were held out for prediction purposes. Data plotted as red and yellow circles correspond to leave-one-phantom cross-validation predictions and temperatures predicted for the left-out phantom spectra in the prediction data set, respectively. Dashed lines represent the ideal unity line.

Results for the leave-one-phantom-out process are presented in Figure 6 and Table 2. The series of temperature correlation plots in Figure 6 present model outputs when using the optimum number of latent variables listed in Table 2. In each case, temperature predictions for the calibration points align well with the unity line and are consistent with the SECV values listed in Table 2, coupled with low bias and strong R^2^ values. Across these seven calibration models, the SECVs range from 0.20–0.26 °C.

Prediction results are mixed for these models. In the best cases, predicted temperatures match the assigned temperatures and the SEP values are similar to the corresponding SECVs. Specifically, the models obtained when Phantoms 5, 6, and 7 were held out have similar SEP and SECV values and the R^2^ values indicate a strong correlation between the predicted and assigned temperatures. When Phantoms 2, 3, and 4 were held out, on the other hand, the SEP values were higher than the SECVs, but the correlation between the predicted and assigned temperatures remains strong, as indicated by R^2^ values between 0.96–0.97. Such findings suggest a prediction bias for these models, which is verified by the calculated bias values listed in Table 2.

The poorest performing model was obtained when the data from Phantom 1 were held out. In this case, the SEP is a factor of 3.25 higher than the SECV, the R^2^ is 0.67, and the bias is −0.20. Inspection of Figure 6A reveals a grouping of temperature predictions between assigned temperatures of 23 and 24 °C, where the trend in the predicted temperatures is nearly orthogonal to the unity line. Inspection of Figure 3 over this temperature range for Phantom 1 uncovers a series of spectral measurements that deviate from the trend, suggesting a systematic error corresponding to variations at the interface between the phantom and the sensing module. These points were not identified as outliers in our analysis described above, and therefore were retained in this dataset.

Despite the observed biases and systematic deviations in temperature predictions, the cumulative results presented in Figure 6 and Table 2 strongly support the analytical utility of near-infrared spectroscopy to predict temperatures within these tissue-simulating phantoms. Our analysis illustrates strong correlations between predicted and assigned temperatures even when the chemical composition of the sample is not represented in the calibration dataset.

Measurement biases are evident from the leave-one-phantom-out analysis, particularly when Phantoms 2, 3, and 4 are left out. Both positive and negative biases were observed for the analysis of these three phantoms (see Table 2). Differences in the absorption and scattering properties of the phantom matrixes could potentially contribute to spectral offsets, and thereby contribute to the observed concentration biases. If matrix composition were responsible here, a trend in the magnitude of the bias would be observed with respect to the amount of water in the phantom matrix. Such a trend is not observed, which suggests phantom composition is not responsible for the offsets. For this reason, it is likely that variations in the placement of the sensing module on top of the sample surface are principally responsible for the observed offsets.

## 4. Conclusions

The PCA analysis highlights two important features of the raw spectral data. First, systematic variance is evident in a set of PCA score–temperature plots (Figure 2, Figure 3, and Figure S1), corresponding to periodic instabilities of the phantom-to-spectrometer interface used for data collection. These regions of interface instability resulted in the removal of 48 outlier spectra. Second, slopes are evident in the score–temperature plots, thereby providing direct evidence of sensitivity to temperature embedded within the near-infrared spectra captured within these datasets. A corresponding set of linear regression analyses reveals a strong correlation between spectral factors and sample temperature, which is the basis of the PLS calibration models. The source of offsets observed in these score–temperature plots is not identified and is likely related to a combination of absorption/scattering variations associated with the composition of the different phantoms, as well as uncontrolled variations associated with the sample interface, instrumentation, and ambient environment. 

Temperature measurements are demonstrated by using two PLS calibration strategies. In both cases, analytical utility was based on prediction accuracy from spectra collected outside the calibration period. Firstly, the spectral data collected for each phantom were split by placing the data collected during the first three temperature values into a combined calibration dataset, and the data collected during the final two temperature steps were placed into a combined prediction dataset. Secondly, a series of seven PLS calibration models were generated, each with all spectra for one phantom removed from the calibration dataset and used solely for prediction purposes. The SEP is 0.22 °C in the first case. For the leave-one-phantom-out PLS models, the SEP ranges from 0.19–0.65 °C, with evidence of interface instability for the higher SEP results. SEP values are less than 0.5 °C for five of the seven leave-one-phantom-out PLS models, with evidence of interface variance in the two higher cases. 

It is important to recognize that omitting, from the calibration dataset (training set), all data from the prediction phantom represents a high bar for the calibration function. Results obtained from the first strategy of splitting all data into calibration and prediction datasets suggest that standard multivariate methods might reduce errors for the leave-one-phantom-out PLS models.

Wellness applications, such as monitoring a person’s basal temperature, provide insights into the body’s ability to recover from activity and may be useful in understanding a person’s susceptibility to illness or disease. Fitness-related applications may provide critical information regarding the onset of heat-related illness associated with rigorous exercise or exercise in extreme heat and the body’s corresponding ability to compensate through heat-dissipation mechanisms. While body temperature is a tightly regulated parameter in the maintaining of homeostasis, a typical operating range of 35–40 °C is nestled between the boundaries of hypo- and hyper-thermia.

Although these measurements were performed below physiological values, the findings published by others [12,13,14,15] demonstrate that the temperature sensitivity of the spectral features of water can be used effectively to predict temperatures over a wide range. It is expected that the temperature-sensitive shifts in the underlying water spectrum used for these measurements will also be useful when extending the measurements to physiological temperatures. 

Overall, the in vitro results presented here support the continued development of near-infrared spectroscopy for noninvasive body temperature measurements. Results from two types of PLS analyses illustrate that standard errors of 0.2–0.3 °C are attainable with straightforward PLS calibration models in a tissue-simulating phantom. These prediction errors are promising in that they are lower than a targeted uncertainty of ±0.5 °C deemed required for real-time wellness and fitness applications. 

Going forward, future gelation phantom studies must be based on formulations that permit temperatures that cover our targeted physiological range (35–40 °C). These findings also underscore the need to enhance the stability of the interface hardware to permit human studies with prototype sensors. Additional sources of spectral variance are expected for a wearable format, including movement artifacts as well as differences in the anatomy of the skin sample. These additional variations will demand improved hardware designs coupled with more sophisticated data processing methods to realize stable, reproducible coupling between living skin and the sensing module.

## Figures and Tables

**Table 1:** Composition and temperature profiles for tissue-simulating phantoms ^1^.

Phantom	Gelatin (%)	Intralipid (%)	Water (%)	Temperature Step Profile (°C)
1	28.1	9.4	62.5	24, 20, 21, 22, 23
2	26.6	9.2	64.1	24, 23, 20, 21, 22
3	25.9	9.9	64.2	20, 23, 21, 24, 22
4	25.4	9.0	65.6	24, 20, 21, 23, 22
5	22.2	9.7	68.1	20, 21, 22, 24, 23
6	19.4	11.1	69.5	23, 20, 22, 24, 21
7	19.0	9.8	71.1	23, 20, 24, 21, 22

^1^ Percentages listed as weight-percent.

**Table 2:** Results from leave-one-phantom-out PLS models.

Held Out Phantom	Latent Variables	SECV (°C)	SEP (°C)	RSEP (%)	R^2^ (Calibration)	R^2^ (Prediction)	Prediction Bias (°C)
1	2	0.20	0.65	2.86	0.97	0.67	−0.20
2	3	0.24	0.34	1.51	0.95	0.96	0.21
3	1	0.26	0.57	2.46	0.95	0.97	−0.54
4	1	0.26	0.35	1.56	0.95	0.97	0.31
5	1	0.25	0.27	1.19	0.95	0.95	0.04
6	1	0.24	0.29	1.27	0.95	0.93	−0.09
7	1	0.26	0.19	0.84	0.94	0.99	0.11

## Data Availability

The datasets presented in this article are not readily available due to commercial restrictions. Requests to access the datasets should be directed to contact@rockleyphotonics.com.

## Supplementary Materials

The following supporting information can be downloaded at: https://www.mdpi.com/article/10.3390/s24123985/s1. Figure S1: Depiction of the PCA score–temperature plots from Figure 2 while highlighting the data points selected for removal from the overall dataset due to instabilities in the coupling between the surface of the phantom and the sensing module. Table S1: Linear regression results for principal component scores versus temperature plots. Table S2: Linear regression results for scores versus phantom component concentration.
