# Supplementary material for: Noninvasive Temperature Measurements in Tissue-Simulating Phantoms Using a Solid-State Near-Infrared Sensor
Source: Sensors (Basel). 2024 Jun 19;24(12):3985. doi: 10.3390/s24123985 (PMC11207550; doi:10.3390/s24123985)
Supplement: Supplementary file 1 [file sensors-24-03985-s001.zip › sensors-3015567-supplementary.pdf]

sensors

MDPI

Supplemental Information

Noninvasive Temperature Measurements in Tissue Simulating Phantoms using a Solid-State Near-Infrared Sensor

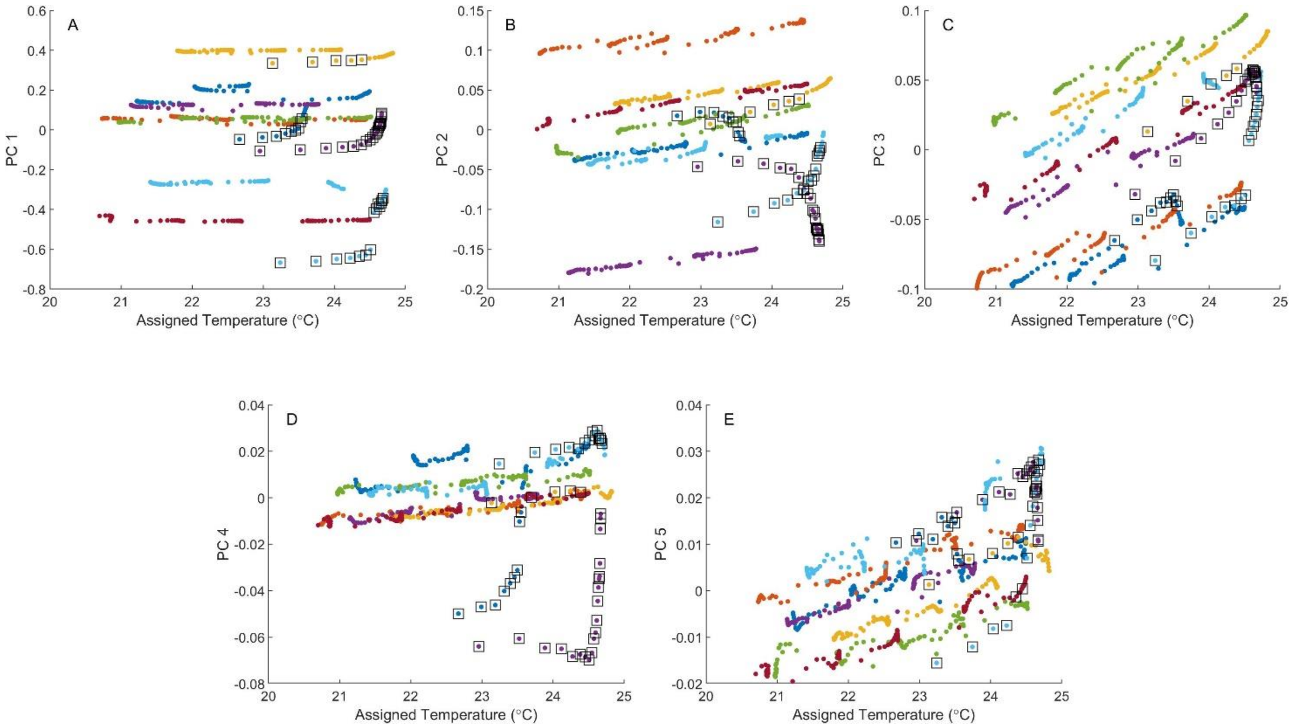

Figure S1. Depiction of the PCA score-temperature plots from Figure 2 while highlighting the datapoints selected for removal from the overall dataset due to instabilities in the coupling between the surface of the phantom and the sensing module.

Table S1. provides results from a linear least-squares fit to each of the phantom datasets presented in the PCA scores – temperature plots in Figure 2. These results show measurable correlations between these PCA scores and temperature for the 2nd, 3rd, and 4th principal components. Table S1. Linear regression results for principal component scores versus temperature plots *.

Phantom	1	2	3	4	5	6	7
Data points	91	100	95	80	100	86	100
				Principal Component 1			
β_1^a	-0.4 ± 4	-4.1 ± 0.7	-0.4 ± 0.7	3.9 ± 0.8	0.5 ± 0.6	-7 ± 1	-2.3 ± 0.5
β_0^b	0.18 ± 0.09	0.13 ± 0.02	0.48 ± 0.02	0.03 ± 0.02	-0.07 ± 0.01	-0.11 ± 0.03	-0.41 ± 0.01
R^2	0.0001	0.24	0.18	0.23	0.39	0.23	0.18
				Principal Component 2			
β_1^a	9.8 ± 0.3	10.4 ± 0.3	1.0 ± 0.3	11.6 ± 0.2	15.4 ± 0.6	12.4 ± 0.5	13.2 ± 0.2
β_0^b	-0.250 ± 0.007	-0.127 ± 0.007	-0.172 ± 8	-0.426 ± 0.004	-0.35 ± 0.01	-0.32 ± 0.01	-0.276 ± 0.004
R^2	0.92	0.92	0.90	0.98	0.89	0.87	0.99
				Principal Component 3			
β_1^a	17.0 ± 0.6	17.2 ± 0.4	18 ± 4	20.3 ± 0.4	20.1 ± .04	18.2 ± 0.7	20.5 ± 0.3
β_0^b	0.47 ± 0.01	-0.454 ± 0.009	-0.374 ± 0.008	-0.47 ± 0.01	0.40 ± 0.01	-0.39 ± 0.02	-0.046 ± 0.007
R^2	0.91	0.95	0.97	0.97	0.96	0.88	0.98
				Principal Component 4			
β_1^a	-5.8 ± 0.4	-5.3 ± 0.1	-6.6 ± 0.2	-6.5 ± 0.2	-4.3 ± 0.2	-9.3 ± 0.5	-6.5 ± 0.1
β_0^b	0.12 ± 0.01	0.120 ± 0.003	0.160 ± 0.004	0.151 ± 0.004	0.103 ± 0.003	0.20 ± 0.01	0.156 ± 0.003
R^2	0.66	0.94	0.95	0.96	0.89	0.82	0.96
				Principal Component 5			
β_1^a	0.06 ± 0.3	0.4 ± 0.1	0.67 ± 0.08	-0.5 ± 0.2	1.12 ± 0.08	0.3 ± 0.1	0.87 ± 0.08

β_0^b	-0.010 ± 0.008	-0.004 ± 0.002	-0.011 ± 0.002	0.017 ± 0.004	-0.036 ± 0.002	-0.002 ± 0.002	-0.021 ± 0.002
R^2	0.0004	0.13	0.40	0.08	0.66	0.11	0.56

* Analysis performed after removing outlier spectra. [a] Slope units = (1/°C) x 10^3; [b]Y-intercept units = unitless.

Table S2. summarizes the linear regression results for plots of PCA scores versus the amount of each chemical constituent used to construction the seven phantoms. These analyses were performed on the individual phantoms as shown in Figure 4. These results reveal that the PCA scores for the 1st principal component is mostly correlated with the concentrations of water and gelatin in the phantom matrixes. Table S2. Linear regression results for scores versus phantom component concentration.

Principal Component	1	2	3	4	5
Water					
β_1^a	-7.7 ± 0.2	-0.2 ± 0.1	0.83 ± 0.06	0.06 ± 0.06	0.010 ± 0.009
β_0^b	5.1 ± 0.1	0.14 ± 0.07	-0.05 ± 0.04	-0.042 ± 0.009	0.007 ± 0.006
R^2	0.73	0.007	0.21	0.03	0.002
Gelatin					
β_1^a	6.7 ± 0.2	0.14 ± 0.09	-0.79 ± 0.05	-0.03 ± 0.01	0.004 ± 0.008
β_0^b	-1.60 ± 0.04	-0.03 ± 0.02	0.19 ± 0.01	0.008 ± 0.003	-0.001 ± 0.002
R^2	0.70	0.004	0.24	0.01	0.0004
Intralipid					
β_1^a	-19 ± 2	0.8 ± 0.5	4.1 ± 0.3	-0.56 ± 0.07	0.12 ± 0.04
β_0^b	1.9 ± 0.2	-0.08 ± 0.05	-0.40 ± 0.03	0.054 ± 0.007	-0.012 ± 0.004
R^2	0.19	0.004	0.21	0.10	0.01

[a] Slope units = (1/conc) x 10^2; [b]Y-intercept units = unitless.
